# A meta-analysis of the effects of glucagon-like-peptide 1 receptor agonist (GLP1-RA) in nonalcoholic fatty liver disease (NAFLD) with type 2 diabetes (T2D)

**DOI:** 10.1038/s41598-021-01663-y

**Published:** 2021-11-11

**Authors:** Samit Ghosal, Debasis Datta, Binayak Sinha

**Affiliations:** 1 Nightingale Hospital, Kolkata, India; 2 Fortis Hospital, Kolkata, India; 3 AMRI Hospitals, Kolkata, India

**Keywords:** Endocrine system and metabolic diseases, Non-alcoholic fatty liver disease

## Abstract

Treatment options for nonalcoholic fatty liver disease (NAFLD) and type 2 diabetes (T2D), two conditions which coexist, are limited though weight loss is an important strategy to improve outcomes in either disease. Glucagon-like peptide 1 receptor agonist (GLP1-RA) present a novel option to treat this dual disease by their salutary effects on glycaemic control and weight reduction. Eight randomized controlled trials on T2D and NAFLD from the Cochrane Library, Embase, and PubMed were included in this meta-analysis. The Comprehensive Meta-Analysis Software version 3 was used to calculate the effect size. In a pooled population of 615 patients—297 on GLP1-RA and 318 in the control arm, GLP1-RA produced a significant improvement in alanine aminotransferase [standardised mean difference (SDM), − 0.56, 95% CI − 0.88 to − 0.25, P < 0.01], aspartate aminotransferase (SDM, − 0.44, SE, 95% CI − 0.64 to − 0.24, P < 0.01), gamma glutaryl transaminase (SDM, − 0.60, 95% CI − 0.86 to − 0.34, P < 0.01) and reduction in liver fat content (LFC) (SDM, − 0.43, 95% CI − 0.74 to − 0.12, P < 0.01), as well as glycosylated haemoglobin (SDM, − 0.40, 95% CI, − 0.61 to − 0.19, P < 0.01) and weight (SDM, − 0.66, 95% CI, − 0.88 to − 0.44, P < 0.01), in comparison to standard of care or placebo. Significant improvement in biopsy resolution was also seen in the GLP1-RA arm (Rate Ratio, 6.60, 95% CI 2.67 to 16.29, P < 0.01). This is possibly the first meta-analysis conducted exclusively in patients with T2D and NAFLD which presents a strong signal that GLP1-RA, improve liver function and histology by improving glycaemia, reducing body weight and hepatic fat, which in turn reduces hepatic inflammation.

Trial Registration: PROSPERO (ID: CRD42021228824).

## Introduction

Non-alcoholic fatty liver disease (NAFLD) and type 2 diabetes (T2D) coexist frequently, sharing a common pathophysiological thread of central obesity, insulin resistance, disordered fat deposition and activation of inflammatory cascades which lead to poor outcomes in both these conditions, synergistically^1^. NAFLD related deaths are common in type 2 diabetes (T2D) and deaths due to cirrhosis in type 2 diabetes are common^1,2^. Strategies addressing the joint burden of NAFLD and T2D are therefore a need of the hour since effective treatments for this dual threat are few and far between.

Weight loss results in improved outcomes in both T2D and NAFLD^3^. This has traditionally been addressed by lifestyle changes, which has its own limitations. In the recent past, the introduction of glucagon-like peptide 1 receptor agonist (GLP1-RA), a group of anti-hyperglycaemic agents, which work on the incretin axis and improve insulin secretion while lowering glucagon secretion from the pancreas in the therapeutic armamentarium of T2D, has resulted in improved cardiovascular outcomes along with improved metabolic control and significant weight reduction^4^. Naturally this presents an attractive strategy for managing the joint burden of T2D and NAFLD.

Currently there are five GLP1-RA approved for clinical use. Exenatide and lixisenatide are derived from exendin, while liraglutide, albiglutide, dulaglutide and semaglutide are derived from native human GLP1. Lixisenatide is short acting with a short half-life. Exenatide is available in both short and long-acting forms. Liraglutide is intermediate acting while the others are long acting, the duration of action naturally related to their half-lives. In clinical trials both liraglutide and dulaglutide have similar efficacy and weight reducing potential and are slightly superior to lixisenatide and exenatide. However, semaglutide seems to be the most powerful agent both in terms of glycaemic lowering and weight loss, in clinical trials thus far^4^.

However, there is a distinct lacuna of data supporting the use of GLP1-RA in T2D with NAFLD. The phase 2 Liraglutide Efficacy and Action in Non-Alcoholic Steatohepatitis (LEAN) study included 52 patients randomly assigned to liraglutide (n = 26) or placebo (n = 26)^5^. Nine of the 23 (39%) patients on liraglutide who completed the study showed a resolution of NASH at repeat biopsy compared with 2 of 22 (9%) patients on placebo. The results though were far from homogenous with multiple dropouts and worsening of hepatic fibrosis in 2 patients, with the histological effect losing significance when corrected for weight loss. A commentary on this paper suggested that “ideally, we would like to see an agent for NASH that shows improvement in fibrosis and NAFLD activity score (NAS), not just a lack of worsening of these endpoints”^6^. A recent phase 2 study on patients with NASH showed that treatment with semaglutide, another GLP1-RA, resulted in significant resolution of NASH when compared to placebo^7^. However, semaglutide use was not associated with a reduction of hepatic fibrosis. In addition, a meta-analysis of the LEAD trials with liraglutide showed a reduction in ALT levels with Liraglutide in a dose of 1.8 mg, which were not replicated with lower doses and were not statistically significant when adjusted for body weight^8^.

Phase 3 studies with other GLP1-RA have also been restricted by small sample sizes, differing and heterogenous end points and short durations^5,7,9–14^. Therefore, awaiting publication of randomized controlled trials which are in the pipeline, a robust meta-analysis was conducted with a large sample size to illustrate the effects of GLP1-RA in patients with T2D and NAFLD, exclusively, to explore the impact of an additional medication to treat this twin threat.

## Materials and methods

This meta-analysis was conducted according to the recommendations of the PRISMA statement and registered with PROSPERO (ID: CRD42021228824)^15^.

### Literature searches, search strategies and eligibility criteria

The randomized prospective studies were identified through a thorough database search (Cochrane Library, PubMed, and Embase), which included the MeSH terms “type 2 diabetes”, “liraglutide”, “exenatide”, “alanine transaminase”, “aspartate aminotransferases”, and “Non-alcoholic fatty liver disease.” Primary search was divided into three categories: (a). Related type 2 diabetes (“T2DM”, and “type 2 diabetes mellitus”), (b). Related to the pharmaceutical agent of interest (“GLP1-RA”, “(Glucagon like peptide 1 receptor agonist”, “liraglutide”, “lixisenatide”, “exenatide”, “exenatide LAR”, “dulaglutide”, “albiglutide”, “semaglutide”, and (c). Hepatic outcomes (“NASH”, “Non-alcoholic steatohepatitis”, “Non-alcoholic Fatty Liver Disease”, “Alanine Transaminase”, “ALT”, “Aspartate Aminotransferases”, “AST”, “Gamma-glutamyl transferase”, and “GGT”). Furthermore, the primary search filters included human data and clinical trials, although no search restrictions on time or language were used. While performing the Cochrane library search, the outcome keywords [(a), (b), and (c)] were clubbed using Boolean OR. The search results were then combined using Boolean AND to yield the first set of citations. The initial search was followed up by a detailed manual search filtering the duplicates and selecting those that met the predetermined inclusion criteria. Any citation that compared GLP1-RA versus a control arm was included for analysis.

### Data extraction including assessment of quality of studies

All the authors independently conducted a web-based search for relevant citations dependent on the selected keywords. Additional filters included a cap on age above 18 years and clinical trials. No restrictions were placed based on language or date of publication. SG conducted the meta-analysis. Any disagreements were resolved by conducting additional independent searches on a different day.

Having identified the eight citations to be taken up for analysis, data required for both primary and secondary analysis were entered into an Excel sheet. The chances of any error in entering the data were cross-checked by another author (DD). The quality of the selected citations was assessed using the Cochrane risk-of-bias algorithm, which included random sequence generation, allocation concealment, blinding of participants and personnel, blinding of outcome data, incomplete outcome data, selective reporting, and other biases. (Supplementary Fig. S1) All the selected citations were evaluated along with their supplementary data and scored individually by BS & SG. Any dispute was reassessed by DD, and a final decision was taken by consensus. Individual publication bias was analysed using funnel plots. (Supplementary Fig. S2) Since a minimum of three rows are required to construct a funnel plot, the biopsy resolution studies could not be assessed for bias (only two studies reported biopsy results).

After the initial process, a manual search was conducted jointly to identify the citations that met the inclusion criteria:

- Randomized controlled trials.
- Age limit: 18–75 years, with type 2 diabetes mellitus and documented NAFLD.
- Inclusion of a control arm not documented to make any impact on hepatic outcomes.
- A minimum of 12 weeks of follow-up.
- Reporting of at least two hepatic outcome measures, one inflammatory and another structural in nature.
- Reporting of metabolic outcomes: glycosylated haemoglobin (HbA1C), serum triglycerides (TG), body mass index (BMI), and body weight.
- A clear documentation of exclusion of all non-NAFLD related hepatic dysfunctions.

**Figure 1:**
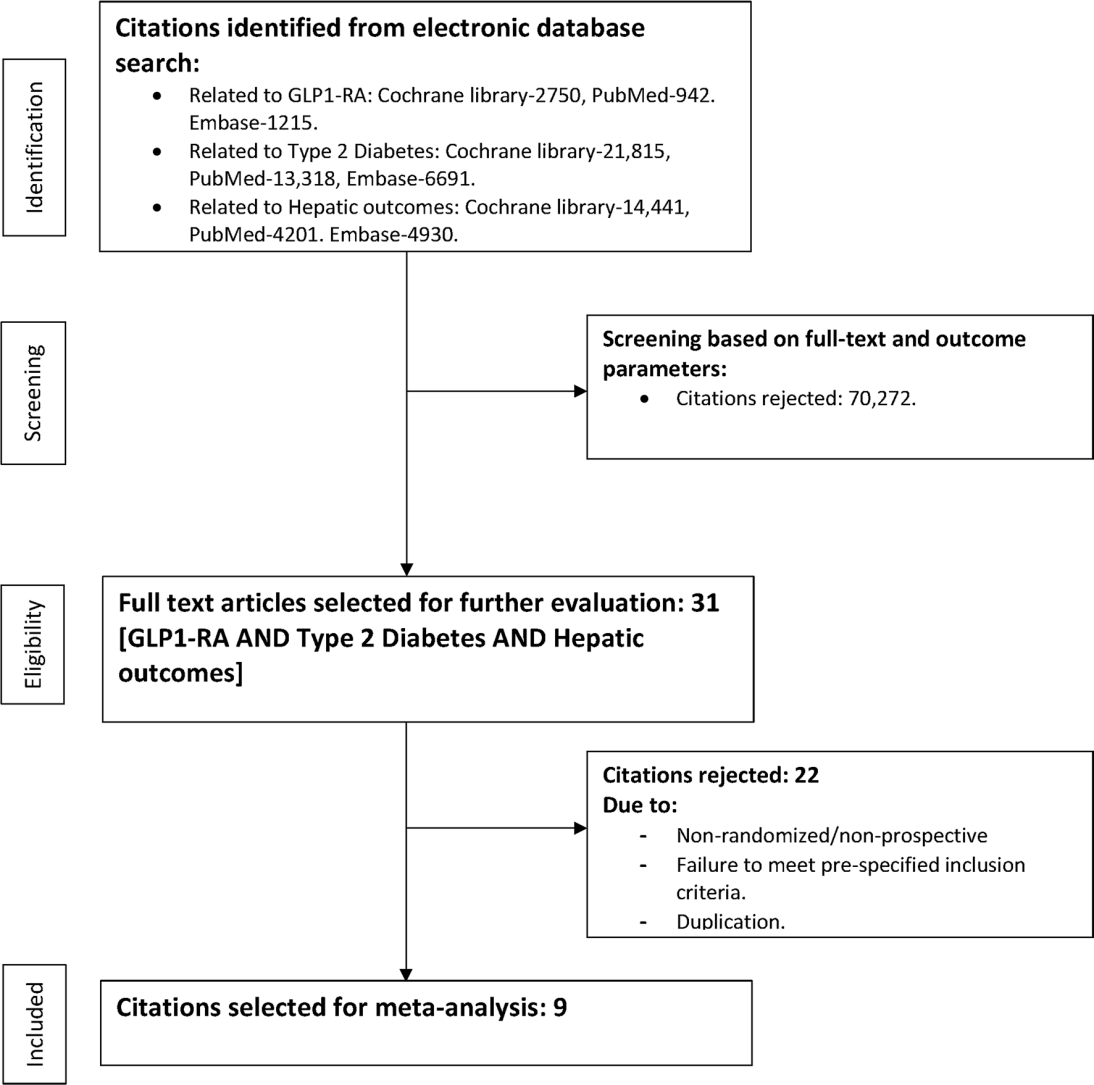
Study selection process.

The process of data extraction is detailed in Fig. 1.

### Patient approval and clearance from the ethical committee

In view of being a systematic review and meta-analysis, there was no direct handling of patients. In addition, effect size estimates that were already published and in open web-based domains were used to conduct the meta-analysis. As a result, there was no requirement for patient or ethical committee consent.

### Statistical analysis

Standardized mean difference (SMD) was used as the preferred parameter of interest in view of the differing patterns of reporting outcomes of interest in the included citations^5,7,9–14^. Some of the citations reported differences in raw mean without any statistical significance, while others reported the events in both arms only^9–13^. Since four different analytical techniques (Independent groups [difference, p], raw difference [independent groups, CI], independent groups [standard difference], independent groups [sample size, p]) were used to derive the effect size in the citations of interest, standardized mean difference was used to maintain uniformity of reporting^5,7,9–14^. The biopsy resolution parameter was reported as a reduction in rate ratio and hence the final effect size was analyzed using rate ratio. In addition to the effect size, hypothesis testing was performed and reported in the form of 95% confidence interval (95% CI) and p-value. The summary of results was reported in the form of tables which also included the weightage of the individual studies. The analysis was conducted using the comprehensive meta-analysis (CMA) software version 3 (Biostat Inc., Englewood, NJ, USA). Heterogeneity was assessed using the Cochrane Q and Higgin’s I^2^ test, and publication bias was assessed using funnel plots. Heterogeneity was defined as high (> 75%) based on the I^2^ statistic. Depending on the degree of heterogeneity encountered as well as baseline differences in the citations analysed, we chose either the fixed or the random model for analysing effect size.

A sensitivity and subgroup analysis were planned if significant heterogeneity related to the pooled effect size was encountered. In view of the small number of studies included in this meta-analysis, sensitivity analysis using the technique of sequential exclusion to identify the study responsible for high heterogeneity was planned.

A subgroup analysis was planned to use the raw mean difference as the outcomes of choice instead of the standardised mean difference.

### Ethics approval

This is a meta-analysis based on published articles, and hence did not qualify for ethics approval.

## Results

### Baseline characteristics of the studies

The meta-analysis was conducted on a pooled patient population of 615 from 8 citations, divided into 297 individuals on GLP1-RA and 318 on standard of care or an active control (without GLP1-RA). As part of the inclusion criteria, we excluded studies, which included as comparators, anti-hyperglycaemic agents capable of influencing hepatic parameters. Among the eight citations, four (Shao et al., Liu et al., Tang et al., and Tian et al.) had an active control arm^10,11,13,14^. Tian et al. had metformin in the control arm while the other three had insulin. D-LIFT and Dutour et al. had standard of care in their control arm, while LEAN, and Newsome et al. had placebo in the comparative arm^5,7,9^. In the Newsome et al. study, the highest dose of semaglutide (0.4 mg) was selected for this analysis to prevent duplication, since the study had been conducted on three separate doses of semaglutide. The LEAN study was conducted on a pooled population of non-diabetic and T2D patients. Since biopsy resolution was reported separately for those with T2D, we included only the biopsy results for analysis ignoring the markers of hepatic inflammation which were not reported separately for the T2D cohort, to ensure only T2D were assessed in this analysis. The duration of follow up ranged from 12 to 72 weeks. The baseline characteristics of the citations included in the analysis are summarized in Table 1.

**Table 1:** Baseline characteristics of the citations included for analysis.

Studies (Year)[Reference number]	Mean age(years)-GLP1-RA group	Sex (Male/Female)-GLP1-RA group	Total patients (GLP1-RA/control)	Control arm	GLP1-RA arm	Follow up duration
D-LIFT (2020)^9^	46.6 ± 9.1	23/9	32/32	Standard of care without GLP1-RA	Dulaglutide 0.75 mg/week for 4 weeks, then 1.5 mg/week for 20 weeks	24 weeks
Shao et al. (2014)^10^	43 ± 4.1	15/15	30/30	Intensive insulin therapy	Exenatide 5 µg BID for 4 weeks, then 10 µg BID for 8 weeks	12 weeks
Liu et al. (2019)^11^	47.63 ± 10.14	19/16	35/36	Glargine	Exenatide 5 µg BID for 4 weeks, then 10 µg BID for 20 weeks	24 weeks
Dutour et al. (2016)^12^	51 ± 2	13/9	22/22	Standard of care without GLP1-RA	Exenatide 5 µg BID for 4 weeks, then 10 µg BID for 22 weeks	26 weeks
LEAN (2016)^5^	50	18/8	26/26	Placebo	Liraglutide 1.8 mg	48 weeks
Tang et al. (2015)^13^	60.7 ± 16.1	11/7	18/17	Glargine	Liraglutide 1.8 mg	12 weeks
Tian et al. (2018)^14^	58.5 + − 7.6	31/21	52/75	Metformin 1 − 1.5 g/day	Liraglutide 0.6—1.8 mg	12 weeks
Newsome et al. (2020)^7^	54.3 ± 10.2	47/35	82/80	Placebo	Semaglutide 0.4 mg/d	72 weeks

### Outcome measures: liver enzymes

There were six studies which reported ALT and AST, while four reported GGT. The standardized difference in means of change from baseline for ALT (SDM, − 0.56, 95% CI − 0.88 to − 0.25, P < 0.01), AST (SDM, − 0.44, SE, 95% CI − 0.64 to − 0.24, P < 0.01), and GGT (SDM, − 0.60, 95% CI − 0.86 to − 0.34, P < 0.01) were statistically significant (Table 2).

**Table 2:** Effect of GLP1-RA versus control on markers of hepatic inflammation: (a) ALT, (b) AST, and (c) GGT.

Study name	Subgroup within study	Std diff in means	95% CI	p-value	Relative weightage (%)
DLIFT	ALT	− 0.48	− 0.98 to 0.01	0.06	16.65
Tian et al	ALT	− 1.03	− 1.41 to − 0.66	< 0.01	29.14
Tang et al	ALT	− 0.10	− 0.76 to − 0.56	0.76	9.36
Dutour et al	ALT	− 0.10	− 0.68 to 0.50	0.74	11.77
Liu et al	ALT	− 0.48	− 0.96 to − 0.02	0.04	18.47
Shao et al	ALT	− 0.90	− 1.42 to − 0.36	< 0.01	14.61
Combined effect size	ALT	− 0.56	− 0.88 to − 0.25	< 0.01	Heterogeneity (I^2^): 56.89
DLIFT	AST	− 0.45	− 0.95 to 0.04	0.07	16.22
Tian et al	AST	− 0.36	− 0.72 to − 0.01	0.04	31.42
Tang et al	AST	− 0.10	− 0.76 to 0.56	0.76	9.08
Dutour et al	AST	− 0.07	− 0.66 to 0.52	0.81	11.43
Liu et al	AST	− 0.59	− 1.06 to − 0.12	0.02	17.68
Shao et al	AST	− 0.90	− 1.42 to − 0.36	< 0.01	14.18
Combined effect size	AST	− 0.44	− 0.64 to − 0.24	< 0.01	Heterogeneity (I^2^): 14.84
DLIFT	GGT	− 0.58	− 1.08 to − 0.08	0.02	26.98
Dutour et al	GGT	− 0.30	− 0.90 to 0.30	0.32	19.11
Liu et al	GGT	− 0.057	− 1.04 to − 0.10	0.02	29.96
Shao et al	GGT	− 0.90	− 1.42 to − 0.36	< 0.01	23.95
Combined effect size	GGT	− 0.60	− 0.86 to − 0.34	< 0.01	Heterogeneity (I^2^): 0.00

*CI* confidence interval.

### Outcome measures: LFC and Biopsy resolution

Quantitative assessment of liver fat (LFC) was assessed by imaging and in three of the eight citations, whereas only two citations reported biopsy resolution data. The standardized difference in the mean change from baseline for LFC (SDM, − 0.43, 95% CI − 0.74 to − 0.12, P < 0.01), and biopsy resolution (Rate Ratio, 6.60, 95% CI 2.67 to 16.29, P < 0.01) were statistically significant. (Table 3) Since the term biopsy resolution, by itself, signifies a decrease from baseline a negative sign was not used in from of the outcome parameter indices.

**Table 3:** Effect of GLP1-RA versus control on liver fat content (a), and biopsy resolution (b).

Study name	Subgroup within study	Std diff in means	95% CI	p-value	Relative weightage (%)
DLIFT	LFC	− 0.56	− 1.06 to − 0.06	0.03	37.11
Liu et al	LFC	− 0.36	− 0.84 to 0.10	0.12	32.08
Tang et al	LFC	− 0.33	− 1.00 to 0.34	0.34	20.81
Combined effect size	LFC	− 0.43	− 0.74 to − 0.12	< 0.01	Heterogeneity (I^2^): 0.00

Study name	Subgroup within study	Rate ratio	95% CI	p-value	Relative weightage (%)
Newsome et al	Biopsy resolution	6.87	2.64 to 17.88	< 0.01	89.27
LEAN	Biopsy resolution	4.70	0.30 to 74.31	0.27	10.73
Combined effect size	Biopsy resolution	6.60	2.67 to 16.29	< 0.01	Heterogeneity (I^2^): 0.00

*CI* confidence interval.

### Outcome measures: weight, triglyceride (TG), and HBA1c

The standardized difference in means of weight from baseline for weight was in favour of the alternate hypothesis (SDM, − 0.66, 95% CI, − 0.88 to − 0.44, P < 0.01). GLP1-RA was also effective in reduction of HBA1c (SDM, − 0.40, 95% CI, − 0.61 to − 0.19, P < 0.01) and TG (SDM, − 0.22, 95% CI, − 0.42 to − 0.03, P = 0.02) (Table 4).

**Table 4:** Effect of GLP1-RA versus control on metabolic parameters: (a) Weight, (b) HBA1c, and (c) TG.

Study name	Subgroup within study	Std diff in means	95% CI	p-value	Relative weightage (%)
DLIFT	Weight	− 0.66	− 1.16 to − 0.15	0.01	18.28
Liu et al	Weight	− 0.98	− 1.47 to − 0.48	< 0.01	19.08
Shao et al	Weight	− 0.90	− 1.42 to − 0.36	< 0.01	16.42
Tang et al	Weight	− 0.76	− 1.45 to − 0.08	0.02	9.81
Tian et al	Weight	− 0.36	− 0.72 to − 0.01	0.04	36.41
Combined effect size	Weight	− 0.66	− 0.88 to − 0.44	< 0.01	Heterogeneity (I^2^): 22.35
DLIFT	HBA1c	− 0.27	− 0.76 to 0.22	0.28	18.46
Liu et al	HBA1c	− 0.86	− 1.34 to − 0.36	< 0.01	18.94
Shao et al	HBA1c	− 0.50	− 1.01 to 0.02	0.06	16.95
Tang et al	HBA1c	− 0.22	− 0.88 to 0.44	0.52	10.12
Tian et al	HBA1c	− 0.24	− 0.58 to 0.12	0.20	35.53
Combined effect size	HBA1c	− 0.40	− 0.61 to − 0.19	< 0.01	Heterogeneity (I^2^): 17.91
DLIFT	TG	− 0.30	− 0.79 to 0.19	0.23	16.12
Dutour et al	TG	− 0.36	− 0.96 to 0.22	0.22	11.02
Liu et al	TG	− 0.12	− 0.59 to 0.34	0.58	18.04
Shao et al	TG	− 0.50	− 1.01 to 0.02	0.06	14.83
Tang et al	TG	− 0.43	− 1.10 to 0.24	0.20	8.70
Tian et al	TG	− 0.02	− 0.37 to 0.33	0.91	31.28
Combined effect size	TG	− 0.22	− 0.42 to − 0.03	0.02	Heterogeneity (I^2^): 0.00

*CI* confidence interval.

### Sensitivity and sub-group analysis

SMD of ALT was associated with a moderate degree of heterogeneity (I^2^ = 56.89). The major contribution was by the data from Tian et al. Further analysis without this study resulted in a gross reduction in heterogeneity (I^2^ = 22.46). However, the impact on the overall outcomes was still significantly in favor of GLP1-RA (SMD − 0.46, 95% CI − 0.69 to − 0.22, P < 0.01). All the other parameters of interest had either no or negligible heterogeneity and hence sensitivity analysis was not performed.

Subgroup analysis was aimed at analyzing the raw mean difference instead of SMD. The significant impact of GLP1-RA on ALT (− 9.57 U/L, 95% CI − 16.36 to − 2.78, P = 0.01), AST (− 6.47 U/L, 95% CI − 10.66 to − 2.27, P < 0.01), GGT (− 14.37 U/L, 95% CI − 22.86 to − 5.88, P < 0.01), HBA1c (− 0.55%, 95% CI − 0.84 to − 0.25, P < 0.01), and weight (− 2.99 kg, 95% CI − 4.23 to − 1.76, P < 0.01) was retained. However, the impact of GLP1-RA on TG (− 6.75 mg/dL, 95% CI − 21.25 to − 7.75, P = 0.36) differed with no difference in significance compared to the control arm. (Table 5).

**Table 5:** Subgroup analysis of metabolic and hepatic inflammatory markers.

Outcomes	Raw mean difference	95%CI	p-value
**Subgroup analysis**			
ALT	− 9.57 U/L	− 16.36 to − 2.78	0.01
AST	− 6.47 U/L	− 10.66 to − 2.27	< 0.01
GGT	− 14.37 U/L	− 22.86 to − 5.88	< 0.01
HBA1c	− 0.55%	− 0.84 to − 0.25	< 0.01
Weight	− 2.99 kg	− 4.23 to − 1.76	< 0.01
TG	− 6.75 mg/dL	− 21.25 to − 7.75	0.36

## Discussion

### Background information

NAFLD is considered part of the metabolic syndrome and is strongly associated with obesity, T2DM, dyslipidemia and Insulin resistance^16^. Obesity, increased fat content, along with insulin resistance results in liver inflammation leading to fibrosis^17^. Hence, therapies directed in reducing weight, fat content and improving insulin sensitivity should be useful in NAFLD and T2DM patients. GLP1-RA present an attractive strategy therefore for treating this twin threat.

GLP1-RA are hormones secreted by the gut and work on the islet cells increasing insulin secretion while suppressing glucagon^18^. This leads to a reduction in leptin, resistin and monocyte chemoattractant protein-1 and an increase in adiponectin levels which in turn inhibits lipolysis, while reducing fat mass^18^. This translates into a reduction of gluconeogenesis and free fatty acid levels, thereby reducing blood glucose and triglyceride synthesis in the liver, this reduction in liver fat is further augmented by the effects of GLP1-RA in reducing inflammation and apoptosis along with improved tissue remodeling in the liver^18^.

Treatment with Exenatide, a GLP1-RA alleviated steatohepatitis of db/db mice through inhibiting hepatic FFA influx and oxidative stress, suggesting GLP-1 analogue can be a therapeutic option in patients with NASH^19^. Till date however there has been no randomized controlled trial in humans which has addressed this potentially remarkable therapeutic option for a frequently coexisting dual disease. As described earlier, phase 2 results and analysis of liver function derived from trials on T2D assessing glycaemic control, have been restricted by their heterogenous patient population, differing outcomes and short duration. Thus, a meta-analysis assessing the effects of all GLP1-RA in patients with T2D with NAFLD was conducted to address this lacuna which might have far reaching clinical implications.

### Additional information from this meta-analysis

A recently conducted meta-analysis has revealed an improvement in all markers of liver function and structure in patients about 70% of whom had T2D and 30% who did not^20^. Therefore, this is to our knowledge the first meta-analysis on patients with T2D exclusively, conducted on a pooled patient population of 615 patients from 8 studies, with 297 patients on GLP1-RA and 318 patients on standard care or an active control. The follow up period ranged from 12 to 72 weeks. There was significant improvement in ALT, AST, GGT levels in patients on GLP1-RA suggesting improvement in liver inflammation. Quantitative assessment of liver fat, assessed by imaging, also showed significant reduction in liver fat. Meta-analysis of the two studies wherein liver biopsies had been conducted revealed a significant resolution of NASH in patients treated with GLP-1 RA. As expected, GLP-1 RA treatment resulted in significantly improved metabolic control and weight loss.

This meta-analysis indicates that GLP-1 RA is potentially a robust treatment strategy in patients with T2D and NAFLD. As indicated in animal models, this meta-analysis points to the likely reduction in liver fat content which results in decreased levels of free fatty acids and carbohydrates thus reducing the metabolic burden on the liver which translates into alleviation of oxidative stress and liver injury. This in turn improves hepatic inflammation and possibly fibrosis. The significant reduction of HBA1C and body weight are possibly important factors that reduce the inflammatory cascade in the liver by reducing the liver fat content. Reduction in liver enzymes, significant in the meta-analysis, is strongly suggestive of improvement in liver inflammation and oxidative stress. Liver biopsy is the investigation of choice to assess NASH. Meta-analysis of the two studies where liver biopsy was conducted on patients using GLP1-RA provide an improved sample size to confirm the improvement in steatohepatitis, too.

### Limitations and strengths

This meta-analysis has certain limitations. First, data were analyzed from the published effect size and not from individual-level pooled data. This could have resulted in the loss of valuable patient-related outcome information. Second, some of the studies reported mean differences between SGLT-2i and the control arm with its associated statistical significance and CI, while others reported the mean changes in the individual arms only. A few studies did not mention any level of significance. In view of such heterogeneous reporting, an SMD was calculated for this meta-analysis instead of the raw mean difference, which is easier to correlate. Third, the imaging modality used to assess liver architectural changes were not standardized with some studies using ultrasonography while others CT scan, MRI, and even MR spectroscopy. Fourth, although liver biopsy is a gold standard for assessing necroinflammation in the liver, only 2 studies in this analysis provided data on liver biopsy.

The inclusion large number of patients (with NAFLD and T2D), exclusion of studies with agents like pioglitazone in the control arm, which are known to have a positive impact on hepatic outcomes, and inclusion of all RCTs till date were the major strengths of this meta-analysis.

### Conclusion

In addition to lifestyle modifications, we believe GLP1-RA will soon be a useful therapeutic option to manage T2DM patients with NAFLD. Significant improvement in HBA1c and body weight results in reduction in liver fat accumulation, which in turn reduces the hepatic inflammation. This results in improvement of NASH. However, it will be useful to have RCTs with liver biopsy or liver elastography in T2DM patients with NAFLD before and after treatment with GLP1 -RA, as confirmation of these findings. While awaiting results of the RCTs this meta-analysis on a large sample size may well be used to shape guidelines for the treatment of T2D with NAFLD.

## Supplementary Information

Supplementary Information.

## References

[1] Gastaldelli A, Cusi K (2019). From NASH to diabetes and from diabetes to NASH: Mechanisms and treatment options. JHEP Rep..

[2] Muggeo M, Verlato G, Bonora E, Bressan F, Girotto S, Corbelinni M (1995). The Verona diabetes study: A population-based survey on known diabetes mellitus prevalence and 5-year all-cause mortality. Diabetologia.

[3] Vilar-Gomez E, Martinez-Perez Y, Calzadilla-Bertot L, Torres-Gonzalez A, Gra-Oramas B, Gonzalez-Fabian L (2015). Weight loss through lifestyle modification significantly reduces features of nonalcoholic steatohepatitis. Gastroenterology.

[4] Nauck MA, Quast DR, Wefers J, Meier J (2021). GLP-1 receptor agonists in the treatment of type 2 diabetes e state-of-the-art. Mol. Metab..

[5] Armstrong MJ, Gaunt P, Aithal GP, Barton D, Hull D, Parker R (2016). Liraglutide safety and efficacy in patients with non-alcoholic steatohepatitis (LEAN): A multicentre, double-blind, randomised, placebo-controlled phase 2 study. Lancet.

[6] Guss DA, Mohanty SR (2016). Liraglutide’s use in treatment of non-alcoholic fatty liver: An evaluation of the non-alcoholic steatohepatitis study. HepatoBiliary Surg Nutr.

[7] Newsome PN, Buchholtz K, Cusi K, Linder M, Okanoue T, Ratziu V (2021). A placebo-controlled trial of subcutaneous semaglutide in nonalcoholic steatohepatitis. N. Engl. J. Med..

[8] Armstrong MJ, Howlihan DD, Rowe IA, Clausen WHO, Elbrønd B, Gough SCL (2013). Safety and efficacy of liraglutide in patients with type 2 diabetes and elevated liver enzymes: Individual patient data meta-analysis of the LEAD program. Aliment Pharmacol. Ther..

[9] Kuchay MS, Krishan S, Mishra SK, Choudhary NS, Singh MK, Wasir JS (2020). Effect of dulaglutide on liver fat in patients with type 2 diabetes and NAFLD: Randomised controlled trial (D-LIFT trial). Diabetologia.

[10] Shao N, Kuang HY, Hao M, Gao XY, Lin WJ, Zou W (2014). Benefits of exenatide on obesity and non-alcoholic fatty liver disease with elevated liver enzymes in patients with type 2 diabetes. Diabetes Metab. Res. Rev..

[11] Liu L, Yan H, Xia M, Zhao L, Lv M, Zhao N (2020). Efficacy of exenatide and insulin glargine on non-alcoholic fatty liver disease in patients with type 2 diabetes. Diabetes Metab. Res Rev..

[12] Dutour A, Abdesselam I, Ancel P, Kober F, Mrad G, Darmon P (2016). Exenatide decreases liver fat content and epicardial adipose tissue in patients with obesity and type 2 diabetes: A prospective randomized clinical trial using magnetic resonance imaging and spectroscopy. Diabetes Obes. Metab..

[13] Tang A, Rabasa-Lhoret R, Castel H, Wartelle-Bladou C, Gilbert G, Massicotte-Tisluck K (2015). Effects of insulin glargine and liraglutide therapy on liver fat as measured by magnetic resonance in patients with type 2 diabetes: A randomized trial. Diabetes Care.

[14] Tian F, Zheng Z, Zhang D, He S, Shen J (2018). Efficacy of liraglutide in treating type 2 diabetes mellitus complicated with non-alcoholic fatty liver disease. Biosci. Rep..

[15] Ghosal, S., Datta, D. & Sinha, B. *Meta-Analysis of the Effects of Glucagon-Like Peptide 1 Receptor Agonists in Non-alcoholic Fatty Liver Disease Patients with Type 2 Diabetes. PROSPERO 2021 CRD42021228824*. https://www.crd.york.ac.uk/prospero/display_record.php?ID=CRD42021228824.

[16] Godoy-Matos AF, Júnior WSS, Valerio CM (2020). NAFLD as a continuum: From obesity to metabolic syndrome and diabetes. Diabetol. Metab. Syndr..

[17] Chiang DJ, Pritchard MT, Nagy LE (2011). Obesity, diabetes mellitus, and liver fibrosis. Am. J. Physiol. Gastrointest. Liver Physiol..

[18] Gastaldelli A, Marchesini G (2016). Time for Glucagon like peptide-1 receptor agonists treatment for patients with NAFLD?. J. Hepatol..

[19] Yamamoto T, Nakade Y, Yamauchi T, Kobayashi Y, Ishii N, Ohashi T (2016). Glucagon like peptide-1 analogue prevents non-alcoholic steatohepatitis in non-obese mice. World J Gastroenterol..

[20] Mantovani A, Petracca G, Beatrice G, Csermely A, Lonardo A, Targher G (2021). Glucagon-like peptide-1 receptor agonists for treatment of nonalcoholic fatty liver disease and nonalcoholic steatohepatitis: An updated meta-analysis of randomized controlled trials. Metabolites.

