# Supplementary material for: A meta-analysis of the effects of glucagon-like-peptide 1 receptor agonist (GLP1-RA) in nonalcoholic fatty liver disease (NAFLD) with type 2 diabetes (T2D)
Source: Sci Rep. 2021 Nov 11;11:22063. doi: 10.1038/s41598-021-01663-y (PMC8586228; doi:10.1038/s41598-021-01663-y)
Supplement: Supplementary file 1 — Supplementary Information. [file 41598_2021_1663_MOESM1_ESM.docx]

**Title**

**A meta-analysis of the effects of Glucagon-like-peptide 1 receptor agonist (GLP1-RA) in nonalcoholic fatty liver disease (NAFLD) with type 2 diabetes (T2D)**

Samit Ghosal^1^, Debasis Datta^2^, Binayak Sinha^3^

1. Endocrinologist, Nightingale Hospital, Kolkata. India.

2. Hepatologist, Fortis Hospital, Kolkata. India.

3. Endocrinologist, AMRI Hospitals, Kolkata. India

**Corresponding author:** Samit Ghosal

**Email:** ramdasghosal@gmail.com

**Tel:** +91-9674328281

**Supplementary Materials**

**
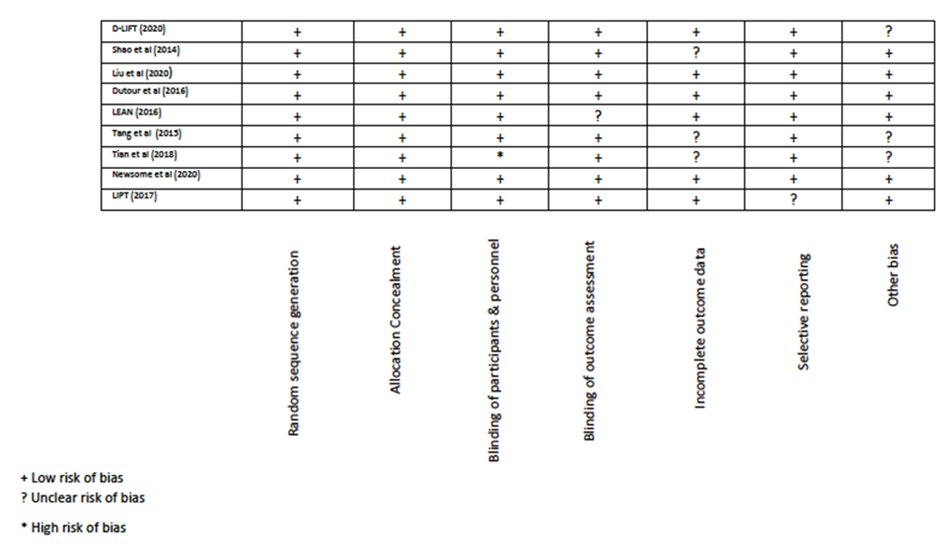
**

Supplementary figure 1: Quality of studies assessed using Cochrane Risk of Bias Algorithm


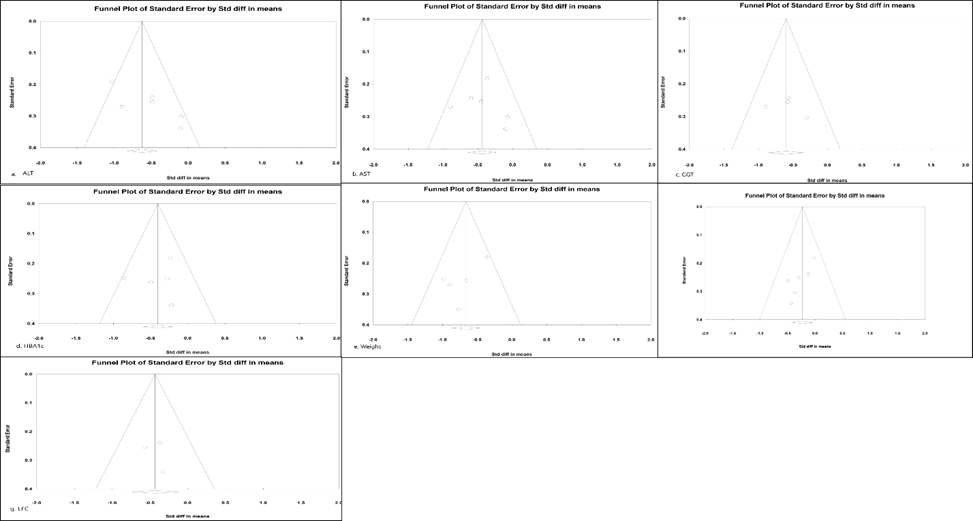


Supplementary figure 2: Funnel plots of individual outcomes, a. ALT, b. AST, c. GGT, d. HBA1c, e. Weight, f. TG, g. LFC
